# Free Amino Acid Alterations in Patients with Gynecological and Breast Cancer: A Review

**DOI:** 10.3390/ph14080731

**Published:** 2021-07-27

**Authors:** Dagmara Pietkiewicz, Agnieszka Klupczynska-Gabryszak, Szymon Plewa, Magdalena Misiura, Agnieszka Horala, Wojciech Miltyk, Ewa Nowak-Markwitz, Zenon J. Kokot, Jan Matysiak

**Affiliations:** 1 Department of Inorganic and Analytical Chemistry, Poznan University of Medical Sciences, 60-780 Poznan, Poland; dagmarapietkiewicz3@gmail.com (D.P.); aklupczynska@ump.edu.pl (A.K.-G.); splewa@ump.edu.pl (S.P.); 2 Department of Analysis and Bioanalysis of Medicines, Medical University of Bialystok, 15-089 Bialystok, Poland; magdalena.misiura@umb.edu.pl (M.M.); wojciech.miltyk@umb.edu.pl (W.M.); 3 Gynecologic Oncology Department, Poznan University of Medical Sciences, 61-701 Poznan, Poland; ahorala@ump.edu.pl (A.H.); ewamarkwitz@ump.edu.pl (E.N.-M.); 4 Faculty of Health Sciences, Calisia University, 62-800 Kalisz, Poland; z.kokot@akademiakaliska.edu.pl

**Keywords:** gynecological cancers, breast cancer, amino acids, proline, metabolomics

## Abstract

Gynecological and breast cancers still remain a significant health problem worldwide. Diagnostic methods are not sensitive and specific enough to detect the disease at an early stage. During carcinogenesis and tumor progression, the cellular need for DNA and protein synthesis increases leading to changes in the levels of amino acids. An important role of amino acids in many biological pathways, including biosynthesis of proteins, nucleic acids, enzymes, etc., which serve as an energy source and maintain redox balance, has been highlighted in many research articles. The aim of this review is a detailed analysis of the literature on metabolomic studies of gynecology and breast cancers with particular emphasis on alterations in free amino acid profiles. The work includes a brief overview of the metabolomic methodology and types of biological samples used in the studies. Special attention was paid to the possible role of selected amino acids in the carcinogenesis, especially proline and amino acids related to its metabolism. There is a clear need for further research and multiple external validation studies to establish the role of amino acid profiling in diagnosing gynecological and breast cancers.

## 1. Introduction

**Figure 1:**
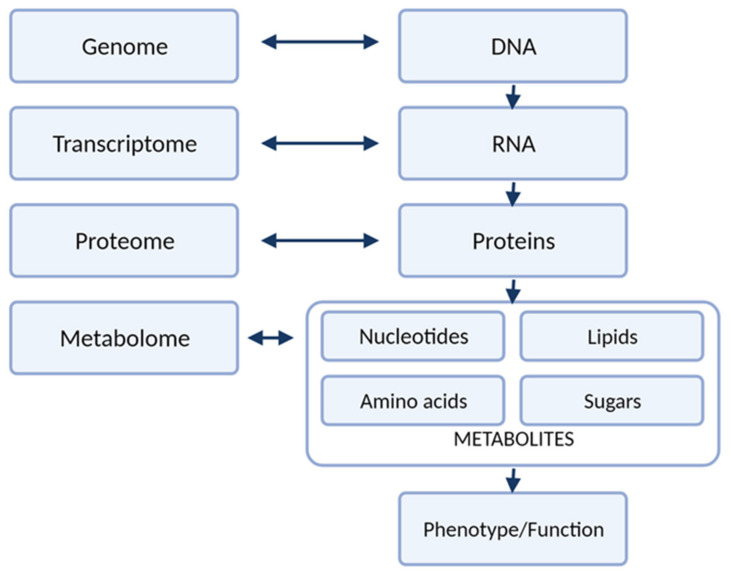
The different levels of “omics” research.

Carcinogenesis is a complex process that affects many pathological pathways. These changes can be investigated on different levels: genes (genome), gene expression (transcriptome), products of mRNA translation (proteome) as well as effects of action of those products (metabolome), as illustrated in Figure 1. With technological advances, cancer biomarker studies evolved from single-protein analysis to profiling of different compounds through the development of high-throughput strategies of genomics, transcriptomics, proteomics, and metabolomics. Metabolomics in an important technique. The metabolome is highly personalized readout of metabolism that is reflective of nature (degraded proteins) and nurture (nutrients, lifestyle choices, the gut microbiome, exposure to drugs and toxins). The unique attribute of metabolomics among the other ‘omic’ technologies is that measuring metabolites provides insight into the biological processes that have occurred, which may be relevant to the health and disease state [1]. Metabolomic studies can be defined as measuring low-molecular-weight metabolites and their intermediates in biological fluids or tissue that reflect the dynamic response to genetic modifications and/or pathophysiological changes due to carcinogenesis. Modern analytical techniques, such as liquid chromatography-mass spectrometry (LC-MS) or nuclear magnetic resonance (NMR) enable fast and comprehensive analysis of metabolites in biological specimens, and detection of thousands of small molecules. In addition, only a small amount of biological specimen is needed for those analyses [2,3].

**Figure 2:**
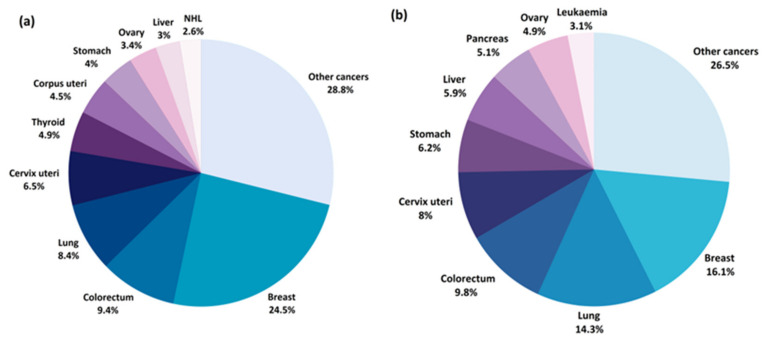
Estimated percentage of new cancer cases (**a**) and cancer deaths (**b**) worldwide for 2020 [8].

Gynecological cancers and breast cancer constitute four out of the eight most common cancers in women globally (Figure 2a) and greatly contribute to women’s morbidity and mortality worldwide [4] (Figure 2b). According to the Global Cancer Observatory data, breast cancer (BC) is the most common cancer in women worldwide, with a crude incidence rate of 58.5/100,000 and mortality rate of 17.7/100,000 [4]. The screening program for early detection of BC are mainly based on mammography, however this examination is reserved for women in postmenopausal age. As many as 20–55% (depending on the country) [5] of breast cancers will develop in women before the age of 50, leaving this population of women without an efficient diagnostic tool. Moreover, imaging diagnostic methods, such as mammography or ultrasound, are time-consuming, require highly strained medical staff to assess the results and require the patient to expose their bodies, which might contribute to a bigger reluctance to adhere to screening. Minimally-invasive and quick biomarker tests from body fluids, e.g., blood, saliva, urine, could be easily accepted by the patient and would open new horizons in early detection of breast cancer in premenopausal women. Among the different gynecological cancers, cervical, endometrial (cancer of the corpus uteri) and ovarian cancers are the most frequent and, thus, considered major women’s health issues. Although cervical cancer (CC) and endometrial cancer (EC) are more common among women, ovarian cancer (OC) is the most lethal [4]. There are no effective methods for preventing or screening for early detection of EC and OC. Currently, about 70% of the patients with OC are diagnosed in advanced stages which is the main cause of its unfavorable prognosis [6]. As for the diagnostic methods, no organized early-detection strategies are available for OC, except for the high-risk groups. The two most commonly used markers of the disease, carcinoma antigen 125 (CA125) and human epididymis protein 4 (HE4), and the risk calculation models that include them, can only be applied to patients with an already detected ovarian mass by other imaging methods, mainly ultrasound, and have been proven to be effective in screening [7]. Early and specific diagnosis of OC is essential to improve the prognosis for patients and reduce mortality, therefore, there is an urgent need for new biomarker discoveries in this field. As far as EC is concerned, the disease is typically symptomatic with abnormal vaginal bleeding or postmenopausal bleeding. The golden standard for diagnosing EC is an endometrial biopsy, an invasive technique of obtaining an endometrial tissue sample for histopathological examination. Early-stage EC markers would certainly help to select and correctly qualify patients from the high-risk group to undergo this biopsy. In addition, novel prognostic markers and markers of pelvic lymph node metastases in EC would significantly help to properly manage EC patients. There are several clinical issues that have not yet been resolved: there is no consensus whether lymphadenectomy should be routinely performed in the intermediate-risk group; there is inconsistent data on the indications for adjuvant treatment (radio-/chemotherapy); and there are no blood markers to easily monitor the effects of treatment and follow-up for a relapse. Metabolomic biomarker research is likely to provide new diagnostic tools that would support clinical decisions and guide the individualized EC care. Primary prevention (vaccinations against oncogenic human papilloma virus types), which greatly contributed to decreasing its incidence in developed countries, is only available for cervical cancer. Nevertheless, it remains a significant health problem for the rest of the world which is reflected by the fact that it is the fourth leading cause of death related to cancers worldwide [4]. Early diagnosis and development of effective screening strategies for women’s cancers are essential to reduce mortality, emphasizing the need for further advancements in these areas.

## 2. Methods

**Figure 3:**
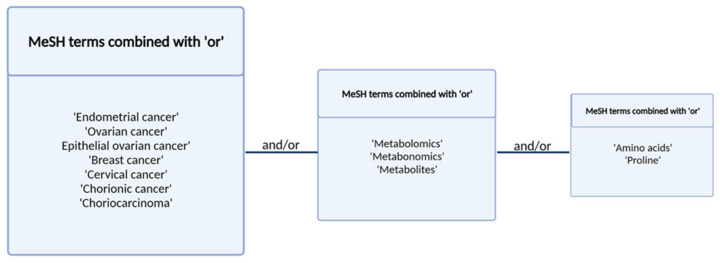
Search terms and strategy.

The authors conducted a systematic, comprehensive literature search using the PubMed database of the studies published from January 2010 to May 2021 with the Medical Subject Headings (MeSH) search terms. The search was conducted using the terms presented in Figure 3. Studies investigating the changes in amino acid profiles of patients with gynecological cancers and breast cancer were included according to the following inclusion criteria:

- original studies;
- studies focused on changes in amino acid profiles in patients with gynecological cancers (endometrial cancer, ovarian cancer, breast cancer, chorionic carcinoma, cervical cancer, vulvar cancer) and breast cancer
- articles in English.

The excluded articles were:

- reviews
- meta-analyses
- letters
- comments
- articles unrelated to the topic
- studies on cell lines or animals

**Figure 4:**
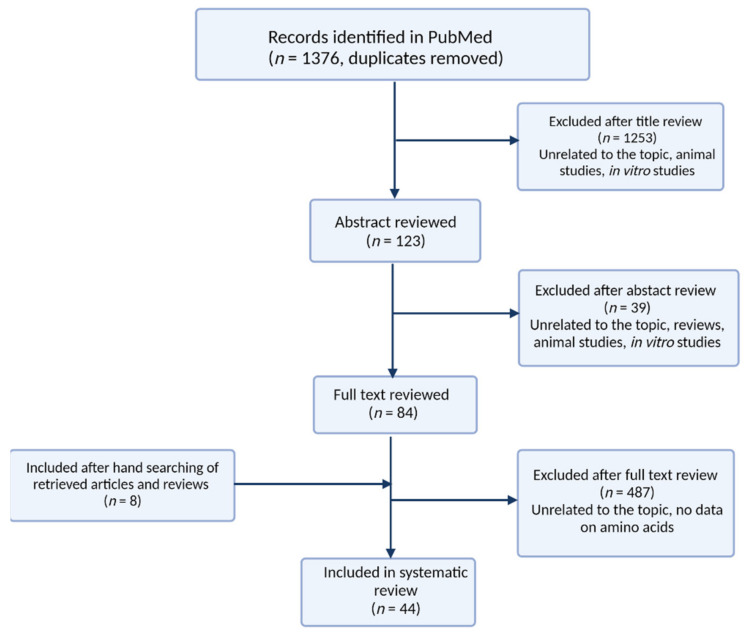
Algorithm of the literature search for reviewed articles.

We screened the titles from the search results for eligibility. Then, each abstract was screened if eligibility was not clear from the title. The last selection was performed on the full text of the selected papers on which a decision could not be made based on the abstract. When studies did not report all the required information but cited relevant studies instead, the related articles were manually screened to check if the additional articles met the eligibility criteria. Finally, additional articles were added to refine this review. The characteristics of the retrieved articles were summarized in Table 1. The number of records produced in PubMed search as well as the screening process was presented in Figure 4.

## 3. Metabolomic Platforms Used for Analysis of Amino Acids

The choice of an analytical technique for metabolomic studies focusing on finding cancer biomarkers depends on the research question and hypothesis, the type of biological samples, the properties of analytes, costs, and availability of expertise. The most commonly used techniques employed in metabolite identification and determination are mass spectrometry (MS) and nuclear magnetic resonance (NMR) [2]. Mass spectrometry techniques are most often supported by various separation techniques, mainly liquid chromatography (LC) and gas chromatography (GC). LC-MS provides more sensitivity compared to NMR and therefore can be used for the analysis of low-abundant metabolites. NMR is distinguished by little to no sample preparation and provides structural and quantitative information on each metabolite [53,54]. In the studies on gynecological cancer, the most prevalent technique for the study of free amino acid alterations is LC-MS. Thirty-one out of forty-four reviewed articles employed different types of LC-MS systems, six of them employed GC-MS, whereas proton nuclear magnetic resonance (^1^H-NMR) was used in seven studies (Table 1). Other analytical approaches used in metabolomics research included in this review included flow-injection mass spectrometry [11,14,16,23,30,32,34,42,46] and direct analysis in real-time mass spectrometry [20].

The analyses in metabolomic studies can be targeted or untargeted. Targeted approaches aim to identify and measure a pre-defined metabolite group, while untargeted metabolomics focus on the identification and relative quantitation of measurable small molecules in a sample with no prior hypothesis [2]. In recent years, great emphasis has been put on global metabolomics. Currently, more analyses are conducted based on specific hypotheses (targeted metabolomics). It should be noticed that in the case of untargeted metabolomics, there is no single method that could be used to analyze the entire metabolome. Therefore, to obtain the broadest coverage of the metabolites present in the analyzed samples, a combination of different analytical methods and methodologies has to be used. Several authors of the articles mentioned in this review used multiple techniques to obtain the widest metabolic profile of the investigated specimens [14,16,23,30,32,34,37,43,46]. Targeted analysis of amino acids is also challenging. Amino acids require derivatization prior to GC analysis due to a lack of volatility of this class of metabolites [55,56]. The application of LC is less cumbersome; however, amino acids are polar metabolites, and therefore they usually have low retention on a standard C18 column [57]. A variety of LC-MS and LC-MS/MS methods were used for targeted analysis of amino acids in the studies on gynecological cancers (Table 1). Some methods employed tandem mass spectrometry without prior chromatographic separation [11,42]. Mass spectrometers separate compounds based on a mass-to-charge ratio and additional chromatographic separation is not always necessary. However, the exceptions are isomeric and isobaric compounds, which are not distinguishable by the mass spectrometer. Isomers among amino acids include such pairs as leucine and isoleucine, alanine and sarcosine, and 1-methylhistidine and 3-methylhistidine. Therefore, in these cases the isobaric species must be separated using chromatography prior to MS detection.

### Validation

The technological progress in metabolomic profiling opens opportunities to discover new cancer biomarkers and elucidate cancer-driven alterations at the metabolomic level. In biomarker research, three key definitions can be distinguished: analytical validity, clinical validity and clinical utility [58]. Analytical validity refers to the assessment of the technical performance of the methodology used for biomarker detection and quantification in the appropriate biological matrix and involves the determination of the method accuracy and precision. Clinical validity implies that the proposed biomarker accurately and reliably detects patients with a disease or is responsible for the separation of the tested cohort into two or more groups based on clinical or biological outcomes. The clinical utility provides the extent to which diagnostic testing (e.g., genetic testing) is useful in facilitating beneficial health outcomes (e.g., preventing mortality, ameliorating morbidity and disability) from interventions that are initiated based on test results. In addition, it provides the information to which the interventions themselves can be applied successfully and cost-effectively. Clinical utility requires high levels of evidence to verify if the biomarker test result is a relevant tool for patient management, making clinical decisions and improving clinical outcomes [58,59].

Validation of the proposed cancer markers is undoubtedly a crucial step in metabolomics studies. Validation of a biomarker candidate on an independent patient cohort or a subset of the first set of samples (after dividing it into training and validation subsets) increases the reliability of the study results. Among the articles discussed in this review, 14 studies included the external validation step and proved the performance of the proposed markers (Table 1). The majority of metabolomic studies on gynecological cancers contain only the discovery step and thus, the obtained findings should be considered as preliminary and require further validation in larger cohorts, preferably in multicenter studies, prior to application in clinical practice. Many small discovery studies, especially those with limited sample sizes, employed internal validation to validate the robustness of the constructed classification models and avoid their overfitting [12,18,20,24,31,39,45]. However, this was only a statistical validation of the created discriminating models performed using the same set of samples. Therefore, the external validation step would be essential to acquire truly unbiased performance estimates. It should also be noted that among the articles selected for the review, there are four studies that aimed to validate biomarkers discovered by other research groups [25,26,30,41]. Since metabolomics is a relatively young field of research, it is anticipated that an increasing number of studies verifying, complementing and improving the previously conducted research will be published in the near future.

## 4. Biological Matrices

In metabolomic studies, we can distinguish two alternate approaches: the top-down and the bottom-up approach [60]. The first one focuses on exploring metabolites, interactions and pathways that are not known a priori [61]. That untargeted research most often concentrates on the evaluation of the complex biological matrix (serum, plasma, urine, etc.), which carries information about the metabolome. The matrix is usually very complex and contains either metabolites and non-metabolic compounds, which can obscure the analytes of interest, and make sample preparation or metabolites detection more difficult (e.g., binding with proteins, matrix effect). It should be emphasized that the complexity of matrix and coexistence of a wide range of metabolites from different chemical classes, might make the simultaneous analysis of all of them impossible due to differences in polarity ionization efficiency or degradation during sample preparation, separation, or in ion source conditions. In turn, the bottom-up approach explores the metabolome basing on previously known metabolites and pathways [62]. In such studies, compounds of interests might be quantified by measuring their levels in a targeted manner. Some of bottom-up studies are based on studies on cell lines, usually cultured in vitro. This approach enables the study of the most basic biological system without potential confounding factors. Hence, studies on cell lines are independent from various lifestyle-related factors: interpersonal variations, sex, age, diet, smoking status, type of analyzed biofluid and its dilution, time of biological matrix collection, etc. On the other hand, cell lines studies have different flaws and limitations. One of the most important issues is the lack of reflection of the complexity of interactions between the cells themselves and between the cells and other levels of structural organization (tissues, organs, systems) [10]. Nevertheless, such studies give insight into basic processes and mechanisms underlying the metabolic pathways. Table 1 summarizes the selection of studies from the last 11 years on changes in the amino acid profiles of patients with gynecological cancers and breast cancer performed on various biological matrices.

### 4.1. Blood Based Matrices

Over the years there has been a number of studies attempting to (1) investigate the human metabolome, (2) find cancer biomarkers, and (3) describe the molecular background of carcinogenesis, that focused on amino acids within various biological matrices. The two most popular matrices used for metabolomic analyses in the reviewed studies were serum (18 studies) [10,11,12,14,15,20,21,22,23,28,29,30,33,35,41,42,43,47] and plasma (17 studies) [9,13,16,24,25,26,32,37,39,40,43,44,45,46,48,50,52]. One study investigated both fluids—serum and plasma [43]—and one other was focused on dry blood spot analysis [44]. The serum is the liquid fraction of full blood and is obtained after clotting and centrifuging the blood sample. To obtain plasma, blood must be collected into an anticoagulant probe and then centrifuged to remove blood cells. According to these studies [63,64], the coverage of the metabolites was similar between serum and plasma. It needs to be highlighted in the amino acids’ investigations that neither of the fluids is superior to the other, and each has some advantages and disadvantages. Subtle variations in metabolites concentrations were revealed due to the blood-based matrix preparation methods [40,52], the effects of time of delay, storage temperature during transportation on the results [65,66,67], and the role of collection tubes [66,67,68]. However, as it was concluded by Breier [65], the amino acids were found to be the least stable class of metabolites among investigated metabolites classes in human serum. Despite the observed influence of the aforementioned factors on the metabolomic results, the authors agree that proper planning and considering these factors during the study design could lead to obtaining reliable and accurate results.

### 4.2. Urine

Among the reviewed papers concerning amino acids, a few studies have been conducted using urine [31,51,69] as a biological matrix to analyze the changes in metabolomes of gynecological and breast cancer patients. Urine has some advantages as its collection is non-invasive, it does not require intervention from the medical staff and is available in large volumes which is worth highlighting as an advantage for studies on cancer. Among the limitations of urine samples is the variable concentration of metabolites quantified in urine. Many factors, such as hydration, perspiration, hormonal homeostasis, environment, diurnal variations, affect the urine volume and may influence the results of metabolomic studies using urine as a matrix. Hence, various approaches are established to eliminate the impact of these factors on analytes concentrations when random spot sampling was applied. The normalization strategies based on appropriate parameters as reference are the most popular. Among others, the creatinine concentration, measurement of the total solute concentration (osmolality), and urine/pure water density ratio (specific gravity) are very common in metabolomic research [70]. Each of these methods has both advantages and limitations. The studies included in the presented review employed different normalization strategies: creatinine value [69] and probabilistic quotient normalization of the metabolite variables using a median calculated spectrum [51]. Thus, normalization strategy should be well thought-out during planning the study. Considering the anatomical continuity of the uterus and lower genital tract, there is a growing interest in searching for biomarkers of gynecological cancer in samples collected in the least invasive way, e.g., urine.

### 4.3. Other Matrices

The role of amino acids in cancer growth and progression is well-established in scientific literature. The analysis of tumor tissue allows observation of the target alteration directly at the tumor site and enables the detection of molecules that could be elusive after secretion into biofluids, the same evaluation of amino acids directly in the tissue emerge as promising matrix for studies on cancer. Seven out of 44 reviewed studies investigated amino acids in tissue samples [17,18,21,23,38,43,49]. Such studies have the advantage of investigating the metabolic alterations at the cancer site and describing the ongoing processes directly related to carcinogenesis. Three among the selected articles investigated tissue samples alongside other matrices [21,23,43]. To thoroughly investigate the local environment at the site of cancer in relation to the systemic metabolic alterations caused by ovarian cancer, Bachmayr-Heyda et al. [23] tested ascites fluid alongside the serum and tissue.

The need to search for new clinically useful methods of early detection of gynecological cancers prompts scientists to investigate more unconventional matrices. The most important cause of this trend is looking for matrices with non-invasive sample collection, independent of confounding factors but providing relevant information about the metabolome complementary to serum, plasma, or urine.

In one study included in the review, research was conducted using cervicovaginal fluid in the search of biomarkers of inflammation, precancerous lesions, or EC [19]. It was found that cervicovaginal fluid appears to be a promising matrix for detecting EC, especially if highly sensitive techniques are used.

The most surprising matrix used in the reviewed studies on amino acids were the nails of breast cancer patients [71]. This matrix can be collected quickly and in a completely non-invasive way. As nails are composed of keratin [71], the material is very stable and, in contrast to urine or blood-based matrices, it is prone to pre-laboratory handling. In the case of nails, even post-mortem specimens can be successfully collected and investigated [72]. According to the cited study [36], nails are a promising matrix for evaluating cancer-related alterations in amino acid metabolism in patients.

## 5. Metabolomic Studies of Gynecological Cancers

The goal of this review was to summarize relevant studies on metabolite-based markers of gynecological and breast cancers. The most widely analyzed cancers in terms of changes in amino acid profiles are BC, EC and OC (Table 1). The available literature concerning amino acid profiles in the less common gynecological neoplasms (vulvar cancer, vaginal cancer and choriocarcinoma) is scarce. In the case of choriocarcinoma only a few studies were available, but they did not meet the eligibility criteria. To the best of our knowledge no studies on vulvar, vagina and chorionic carcinoma that met the eligibility criteria have been published.

## 6. Amino Acid Profile Changes in Gynecological Cancers

Amino acids are an important group of metabolites. Their potential as biomarkers has been highlighted in many studies [73,74,75,76]. They play a key role in many biological pathways, from the biosynthesis of various molecules such as proteins, nucleic acids, enzymes, etc. [77], through serving as an energy source [78] to maintaining redox balance [79]. During tumor progression, the cellular need for free amino acids for DNA and protein synthesis increases, which reduces the plasma concentration of these amino acids. On the other hand, the increased concentration of amino acids in tumors might indicate a high proliferation rate.

The research conducted so far has pointed at various amino acids potentially indicative of cancer, but the evidence is inconclusive. This may be due to using different biological matrices for research, the use of different analytical techniques, or differences in patient groups between studies. Moreover, the diagnostic methods are very often based on the panels of biomarkers consisting of various compounds rather than a single biomarker. Thus, it seems that the biomarker research should not focus on one specific compound but rather take into consideration changes in metabolic pathways involving many different compounds often linked by various relationships.

### 6.1. Breast Cancer

Glutamine is one of the most frequently mentioned amino acids among the proposed biomarkers of breast cancer, as it was previously known that cancer cells consume glutamine to support self-sustained growth and aggressiveness. In the study by Yang et al. [80], the decreased glutamine level in serum and plasma was associated with the accumulation of glutamic acid in the body, enhancing the proliferation of mammary epithelial cells. Neoplastic cell requirements for mitochondrial ATP are lowered, but at the same time, requirements for NADPH and biosynthetic precursors are increased. To maintain the tricarboxylic acid (TCA) cycle, which supports energy production and provides intermediates for other pathways, cancer cells rely on glutaminolysis that produces intermediary metabolites for the TCA cycle, e.g., α-ketoglutarate. Metabolic reprogramming serves cancer cells for utilizing glycolysis, even if oxygen is available. Elevated glucose uptake and lactate secretion rely mainly on dysregulated of pyruvate kinase muscle isozyme M2 (PKM2), however, there are many other known mechanisms. For example, in breast cancer, the xCT antiporter plays the role of amino acid transporter exporting glutamate in exchange for cysteine, a precursor for GSH synthesis [81]. The Warburg effect is also regulated by thyroid hormone (T3) in breast cancer cell lines. T3 stimulates the Warburg effect in invasive triple-negative breast cancer cells [82]. Kus et al. discovered rewiring of the metabolism towards glycolysis and PPP as a hallmark for the late phase of metastasis in vivo while alterations in arginine and polyamines as an early feature of metastatic breast cancer cells [83]. High extracellular lactate level leads to reductive carboxylation of glutamine to citrate in the TCA cycle in breast cancer lines (MCF7 and MDA-MB-231) compared to normal breast cells (MCF10A) [84]. In turn, Gkiouli et al. found that in MCF7 breast cancer cells cultured in media containing limited and standard concentrations of glucose and glutamine, glycolysis, and the TCA cycle were identically driven, suggesting flexibility and nutrient-dependent metabolic direction [85]. Due to the abnormal transport of ammonia to glutamine synthesis, fluctuating levels of alanine and aspartic acid might be observed. Available reports are inconsistent in stating whether the levels of alanine in BC are decreased or increased. According to the studies conducted by Eniu et al. [35], Shen et al. [37], and Yuan et al. [46], alanine levels were decreased. In plasma and serum specimens collected from patients with BC compared with the alanine levels measured in healthy controls. Decreased level of alanine might be caused by alanine flux for gluconeogenesis, which is probably one of the major abnormalities in cancer patients [86]. The increased level of alanine may come from muscle breakdown. Muscles degrade amino acids for energy needs. As a result, the nitrogen is transaminated to pyruvate to form alanine [86]. Amino acid catabolism that focuses on free aromatic amino acids like tryptophan, tyrosine, histidine, and phenylalanine is one of the preferred pathways that support tumor growth. Increased levels of alanine in plasma and tissue samples in patients with BC compared with healthy controls were observed in the studies performed by Budczies et al. [38], Cala et al. [39], and Miyagi et al. [40]. Elevated levels of alanine are most likely caused by increased alanine biosynthesis in response to the increased demand for alanine for TCA cycle. The decreased level of histidine in BC mentioned in a few studies [37,40,43,44,46] may be caused by the increased demand of the cancer cells for histamine [87], that may regulate biological responses related to tumor growth (e.g., migration, angiogenesis, cell invasion) [88,89,90]. Lower concentrations of tyrosine in BC patients are potentially associated with increased tyrosine phosphorylation for ATP synthesis in tumors [35], which is in line with previous studies [90,91]. Only in studies conducted by Li et al. [47] and Wang et al. [44] were levels of tyrosine increased in serum and blood of BC patients. The explanation for the increased tyrosine level might be the fact that deficiency of tyrosine may result in stopping the growth of the BC cells, and the increased tyrosine is the metabolic adaptation to tumor state [44]. All BC studies included in the review pointing to tryptophan as one of the differentiating amino acids consistently indicated a decreased concentration of this amino acid. Previous studies indicated that tryptophan has a role in immunosuppression [92,93]. Kynurenine, which is a metabolized product of tryptophan, may promote differentiation of T regulatory cells, which might help in cancer-mediated evasion of the immune system [94]. The decreased levels of phenylalanine mentioned in a few studies [36,40,46] might be caused by the increased demand for this amino acid by cancer cells [95]. As proline can be used as an energy source and as a precursor for protein synthesis, the need for this amino acid is increased and thus, proline biosynthesis is increased. Research by Li et al. [47] and Miyagi et al. [40] showed the increased level of proline in serum and plasma samples derived from BC patients compared to healthy controls. The remaining studies reported decreased proline concentrations [37,44,45], possibly due to increased proline utilization by tumor cells. Some of them indicated the altered levels of arginine [34,35] and asparagine [34,44,46]. A lower concentration of arginine is associated with reduced anti-tumor response [96]. In the case of asparagine, it has been suggested that it is used by cancer cells to protect cells from apoptosis in case of glutamine deficiency [97]. Moore et al. [41] and Wang et al. [44] proposed cysteine as a BC marker candidate which has three main functions in cancer metabolic adaption: help control oxidative stress, a carbon source for energy production, and production of hydrogen sulfide [98]. These studies indicate the extensive adaptation of metabolism in neoplastic cells to facilitate cancer survival in the case of deficiency of certain compounds.

### 6.2. Cervical Cancer

Amino acids are promising biomarkers of cervical cancer as they have a critical role in cancer growth and are identified among the most differentiating compounds in many metabolomic studies on this tumor. According to Khan et al. [48] aspartate, glutamate and proline levels were significantly higher in patients with CC compared to healthy controls. The mentioned amino acids were associated with an increased risk of developing cervical cancer and were identified as potential biomarkers for early detection of CC [48]. Other metabolomic studies that focused on cervical cancer identified altered levels of various amino acids, e.g., lysine [50], tryptophan [51], methylproline [49] (Table 1). There were many discrepancies in the amino acids proposed as putative cervical cancer markers. Alanine, a precursor of glucose in gluconeogenesis [99], was found at lower concentrations in plasma in patients with cervical cancer compared to healthy controls [52]. Intermediates of TCA cycle (valine and isoleucine) also had lower concentrations in patients with cervical cancer. This observation might suggest a suppression of TCA cycle [94,95]. Abudula et al. [49] observed a significant decrease in alanine level, and a decrease in the levels of tyrosine, phenylalanine, glucose and low-density lipoprotein in tissue samples collected from patients with cervical cancer compared to healthy controls. Those changes are the indicators of increased glycolysis. It has been previously described that this is a typical metabolomic signature of cancer patients [100]. Enhanced glycolysis activity is the main source of energy for cancer. Abudula et al. [49] mentioned that increased glycolysis might be linked to altered regulation of lipid and amino acid metabolism during carcinogenesis. The study performed by Yang et al. [50] was in line with the aforementioned studies. In the spheroids of the cervical squamous cell carcinoma (SiHa cells) model, imitating cancer stem cells, the levels of serine and glutamine were significantly increased. The authors observed the activation of the TCA cycle as a metabolic feature of cancer stem cells, however, the mechanisms for metabolic rewiring are under investigation. As the authors suggest, it may be due to the expression of glycolysis-related enzymes, amino acid transporters, and cell morphology [101]. Lysine biosynthesis, histidine metabolism, and lysine degradation play a very important role in rapid tumor growth. Downregulation of the metabolites involved in the TCA cycle and fatty acid metabolism resulted in rapid but inefficient energy metabolism [50]. The mentioned studies indicate that decreased concentration of amino acids does not necessarily mean an increased protein biosynthesis, but rather an increased energy consumption at the expense of amino acids.

### 6.3. Endometrial Cancer

One of the most frequently proposed differentiating amino acids in EC studies was asparagine [9,11,14,19,59,102]. It is synthesized from glutamine in the presence of asparagine synthetase. Asparagine is an amino donor in urea, purine, and pyrimidine synthesis. It is very common for cancer cells to adapt their metabolic pathways to support their growth and metastases. Asparagine, as well as glutamine, promote cancer progression [103]. Firstly, glutamine is used to produce energy in the TCA cycle [104]. Asparagine serves as a reservoir for cancer cells in the absence of glutamine, protecting cancer cells from apoptosis [105].

Methionine was identified by Ihata et al. [9], Bahado-Singh et al. [14], and Strand et al. [16] as a differentiating amino acid between EC patients and healthy controls. It is a precursor of cysteine, homocysteine, creatine, carnitine, and succinyl-CoA [16]. All studies included in the review indicated decreased methionine concentration in patients with EC compared to controls. Lowering the concentration of methionine leads to changes in the lipid metabolic pathway. In the study performed by Strand et al. [16], an interesting finding was the increased concentration of methionine sulfoxide in patients with short survival. Methionine sulfoxide is the oxidized form of methionine. Its increased concentration under normal conditions has been associated with the aging of the body’s tissues, i.e., the so-called biological aging. Methionine reductase prevents protein oxidation and is also a ROS scavenger [106]. Dysfunction of methionine sulfoxide reductase has been associated with disruption of key signaling pathways, increased tumor cell proliferation, and extracellular matrix degradation [16].

In the study by Troisi et al. [10], one of the three proposed discriminating amino acids was homocysteine. It is a homolog of cysteine and is synthesized from methionine. Homocysteine can be recycled to methionine with the aid of folate. Increased levels of homocysteine are a well-known risk factor for cardiovascular diseases. Upregulated homocysteine and, in consequence, deficiency of folate, vitamin B6, or vitamin B12 involves the transferring of one-carbon groups [107]. In terms of carcinogenesis, this mechanism is potentially associated with DNA synthesis, repair, and methylation [108]. However, the effects of homocysteine on tumor growth remain unclear.

According to the study by Ihata et al. [9], in EC patients, plasma histidine, tryptophan, valine, phenylalanine, asparagine, serine, leucine, and methionine concentrations were lower compared with healthy controls. Only plasma levels of ornithine, isoleucine, and proline were increased in patients with EC. Higher valine levels are generally associated with high body mass index (BMI), and numerous studies have shown that the higher the BMI, the greater the risk of developing EC [109,110,111]. However, results from only two studies [11,18] showed increased levels of valine and were correlated with high BMI. In the rest of the studies [9,10,13], the levels of valine were lower among cases than controls, which suggests that not all obesity-related metabolic alterations imply higher EC risk. Phenylalanine cannot be synthesized in the body, and its deficiency might cause slower cancer growth. Excessive phenylalanine protects cancer cells from starvation and autophagy. Trousil et al. [18] also reported an increased level of proline which may indicate increased proline biosynthesis.

What is interesting in the study of Audet-Delage et al. [15] is that the altered metabolism of serine, threonine, and glycine was pointed out. The metabolism of glycine is connected to the metabolism of serine and threonine [112]. However, decreased concentrations of serine and threonine did not affect the level of glycine. It is possible that this was due to higher levels of N-acetylglycine, which suggests the adaptation of metabolic pathways to support tumor growth.

Tryptophan mentioned in a couple of studies [9,13,17] is associated with the kynurenine pathway. Total tryptophan (free + albumin-bound) has been found to be decreased in some types of cancer (e.g., OC, pancreatic cancer, lung cancer, colorectal cancer). However, free tryptophan is increased in cancer patients [113,114]. In the study where free and total tryptophan were measured simultaneously, tryptophan binding, expressed as % free tryptophan was decreased [115]. The reason for the decreased tryptophan binding is the high concentration of non-esterified fatty acid (NEFA). Tryptophan in plasma can be displaced from its binding site on albumin by NEFA. High concentration of NEFA is observed in cancer patients. The highest the NEFA concentration is the highest amount of free tryptophan is available. Tumors acquire larger amounts of tryptophan probably to achieve immune escape via production of immunosuppressive kynurenine metabolites inducing apoptosis of effector immune [92,116,117]. The results from the study by Suzuki et al. [13] were particularly interesting. In this study the levels of plasma amino acids in patients with EC, OC and CC were compared before and after treatment in the same patients. Decreased levels of tryptophan, histidine, citrulline, and valine were observed in patients with OC before treatment. The levels of those amino acids went up after treatment and were closer to the levels observed in healthy control. The higher concentration of isoleucine before treatment dropped after the treatment. The results of the study support the hypothesis that altered metabolic pathways are caused by cancer (presence).

### 6.4. Ovarian Cancer

Alanine [15,20,22,24,31], histidine [23,25,26,28,29,30], and tryptophan [21,23,25,26,28,32,68] were among the most frequently mentioned differentiating amino acids in the metabolomic studies on OC. Other amino acids that distinguished OC patients from benign ovarian tumors or healthy controls were asparagine, glycine, isoleucine, leucine, methionine, glutamine, citrulline, and valine. Tryptophan levels were decreased in serum, plasma, urine, and tissue samples collected from patients with OC (Table 1). Indoleamine 2,3-dioxygenase which correlates with tryptophan degradation, takes part in suppressing anti-tumor response in the body. Increased degradation of tryptophan by indoleamine 2,3-dioxygenase results in increased kynurenine formation. All these factors, i.e., the activity of indoleamine 2,3-dioxygenase and tryptophan 2,3-dioxygenase, increased kynurenine concentration and increased tryptophan metabolism, potentially lead to increased proliferation of regulatory T-cells that support immunosuppression and thus protect cancer cells [92,116]. The disturbed metabolism of tryptophan leads to the creation of a favorable metabolic microenvironment for the further development of the tumor [25]. Histidine was another proposed potential biomarker of OC in a few studies (Table 1). All studies included in this review demonstrated decreased levels of histidine in OC patients. In the study by Horala et al. [28], decreased histidine correlated with increased levels of histamine. In several studies, it was highlighted that histamine might be a crucial mediator in cancer growth by promoting angiogenesis, cell proliferation and modulating the immune system [118]. In addition, histidine decarboxylase, which converts histidine to histamine, was overexpressed in different types of cancer as well as in OC [107]. The decreased level of histidine was also reported by Plewa et al. [3]. The proposed mechanism responsible for the lowered level of histidine was based on the pathways of nucleotide biosynthesis. The genome instability in early stage of cancer is caused by stress on DNA replication. DNA replication of cancer cells requires the excessive consumption of nucleotides, amino acids and one carbon unit. For the nucleotides’ synthesis, the human body needs one carbon unit, amino acid and carbon dioxide. Histidine is the amino acid involved in the anabolism of one carbon unit. These changes might be responsible for lowered concentration of histidine in cancer patients. Another amino acid proposed as an OC marker, glutamine, is also associated with nucleotide biosynthesis [21,28,33]. Glutamine serves as a nitrogen donor for pyrimidine and purine biosynthesis [119] and provides intermediates for TCA cycle during nucleotides biosynthesis by glutaminolysis as it is the second most essential nutrient after glucose [104,120]. Another possible mechanism of glutamine dependency in tumors is a connection with citrulline. Glutamine is conversed into citrulline which is involved in three metabolic pathways (urea cycle, arginine-citrulline-nitric oxide and arginine biosynthesis). Arginine is known to be involved in various biological pathways like cell proliferation and protein synthesis. It has also been associated with metabolic pathways crucial for carcinogenesis including nitric oxide pathway, and polyamines synthesis that are crucial for DNA and RNA metabolism [121,122]. A commonly observed phenomenon in cancer cells coupling metabolism reprogramming and synthesis of macromolecules is aerobic glycolysis known as the ‘Warburg effect.” Although it provides less ATP (about four molecules per one glucose molecule), cancer cells can produce biomass for rapid proliferation. Various cancer types exhibit a broad range of addiction to glucose, however, ovarian cancer cells can rewire the metabolism and avoid glucose dependency and use glutamine as fuel for cell growth relied on oxidative phosphorylation [123]. Formed spheroids of ovarian clear cell adenocarcinoma (OVTOKO cells) constitute a model for cancer stem cells. The levels of serine and glutamine, as well as adenylates (ATP, ADP, AMP), were remarkably increased in comparison to the 2D OVTOKO model indicating the activation TCA cycle supplying cancer cells with intermediates for biomass [101]. Another study indicates a correlation between uptake and secretion of amino acid and enhanced pyruvate uptake as well TCA cycle. Moreover, it is associated with highly invasive ovarian cancer increasing metastatic potential [124]. Caneba et al. discovered a mechanism underlying nitric oxide (NO)-dependent regulation of the Warburg effect in ovarian cancer cells (OVCA cell line). Under tumorigenic conditions, NO leads to an inhibition of mitochondrial respiration and a decrease in ROS levels via the rise of glutathione and NADPH levels [125]. Methionine is involved in the increased proliferation of cancer cells potentially through the one-carbon metabolism which provides methyl groups for e.g., DNA, amino acids, polyamines [108]. This amino acid in the study by Hilvo et al. [21] was decreased in the serum of OC patients compared to healthy controls and patients with benign ovarian tumors. It may indicate an increased demand for methionine by cancer cells to repair damaged DNA and to reduce oxidative stress. In the paper by Wang et al. [33], methionine level was increased in the serum of patients with OC compared to healthy controls. This might be the case because methionine is a methyl donor in DNA methylation and is a significant metabolite in one-carbon metabolism [108]. Therefore, methionine deprivation might inhibit tumor growth. Proline was suggested as a potential differentiating biomarker in studies of Hilvo et al. [21] and Miyagi et al. [26]. Proline may serve as an energy source in TCA cycle during stress, regulates redox homeostasis and provides signaling reactive oxygen species [126,127].

## 7. Role of Proline Metabolism in Gynecological Cancers

Among 20 DNA-encoded amino acids, proline appears to be unique due to its chemical structure. It is a cyclic and nonessential amino acid containing a pyrrolidine ring which provides its chemical stability [128]. Currently, numerous teams have demonstrated that proline is involved in many biochemical processes playing a significant role as an osmoprotectant, metal chelator, protein chaperone, ROS scavenger, energy source, a regulator of redox balance and NADP+/NADPH level in the cytosol, as well as a signaling molecule in apoptosis/autophagy [127]. Proline metabolism has also been implicated as a metabolic pathway impacted on DNA biosynthesis and epigenetic modifications [129]. Proline synthesis, degradation, and cycling are dysregulated in the microenvironment of cancer cells leading to the impairment of cell proliferation, survival, metastasis, and extracellular matrix function. Here, we discussed the role of proline metabolism as a potential reason for uncontrolled cancer cell growth.

Proline anabolism and catabolism are mainly regulated by the enzymes D1-pyrroline-5-carboxylate (P5C) reductase (P5CR) and proline dehydrogenase/proline oxidase (PRODH/POX) being transferred between mitochondria and cytoplasm. The relationship P5C-proline is known as a proline cycle discovered by Phang and colleagues [130] serving as an engine regulating redox balance. Although it was found a few decades ago, the mechanism underlying the rate of proline cycling is not yet fully understood and includes collagen turnover and activity of PRODH/POX and PYCR as well as prolidase [126]. The synthesis of proline occurs from glutamate by the P5CR (in humans there are three isoforms: PYCR1, 2, L) or proline is converted from ornithine by the ornithine-d-aminotransferase (OAT). The reduction of P5C to proline requires a cofactor, NADPH, which is oxidized in this enzymatical reaction. In turn, the reaction of proline to P5C is catalyzed by PRODH/POX [131] which non-covalently binds flavin adenine dinucleotide (FAD) required for proline oxidation. As a result, it occurs generation of P5C and reduction of flavin followed by spontaneous conversion of P5C into γ-glutamate semialdehyde (GSA). P5C dehydrogenase (P5CDH) using nicotinamide adenine dinucleotide converts GSA to glutamate that can enter the TCA cycle as an α-ketoglutarate. Proline is also converted by OAT into ornithine, a component of the urea cycle [129]. Moreover, proline can be incorporated into collagen molecule, which is a reservoir for proline, since approximately 25% of all its residues constitute prolyl or hydroxyprolyl groups [132]. Due to the presence of proline and hydroxyproline, the triple helix of collagen molecules protects it from undesirable non-specific protease activity. The degradation of extracellular collagen occurs by specific metalloproteinases supplying shorter polypeptides fragments prone to various proteases. At the end, dipeptides containing proline at C-terminal are cleaved by cytosolic prolidase, an enzyme specifically degrading X-proline dipeptide [133]. Approximately, a cell can recover up to 95% of proline from collagen degradation [134].

Of critical importance in cancer research could be the collagen-prolidase-proline axis since recent studies demonstrated several reports for a regulatory role of proline in apoptosis [135]. In terms of gynecological cancers, recent metabolomics studies show increased proline levels [9,18,26,40,47,48]. At the biochemical level, a possible explanation for increased intracellular proline concentration could be the activation of metalloproteinases (MMPs), mainly MMP-2 and -9, causing collagen degradation. Increased collagen breakdown results in (1) increased cancer cell metastatic potential through enhanced mobility and (2) intensive proline release as an alternative energy source [129]. Under conditions of nutrient depletion or hypoxia, proline may serve as an energy source in the microenvironment of cancer cells. The oxidative conversion of one molecule of proline provides around 30 molecules of ATP equivalents [127] supplying energy for the uncontrolled proliferation of cancer cells.

In the context of cancer cells, the demand for sucbstrates for DNA biosynthesis is increased since cells proliferate rapidly and in an uncontrolled way. It demands a remarkable quantity of building blocks for protein and DNA synthesis. Therefore, cancer cell reprograms its function to decrease the rate of collagen biosynthesis and make proline available for PRODH/POX activity [129]. Proline can fuel the production of macromolecules through upregulation of oncogenic factor c-MYC [136] and PI3K [137] signaling pathways. Liu et al. [136] reported that c-MYC upregulation causes activation of proline-supplying enzymes (PYCRs). Proline is not only of great importance for protein synthesis, it is also crucial for the conversion into glutamate or glutamine or indirect support of pentose phosphate pathway (PPP). It is worth mentioning the relationship between the cycling proline and the ratio NADP+/NADPH. Reduction of NADP+ into NADPH is coupled to PPP, a metabolic pathway strongly supporting the generation of purine nucleotides for DNA biosynthesis [121]. Cancer cells facilitate uncontrolled proliferation through reprogrammed cellular metabolism including increased survival capacity under stress conditions. It has been found that HIF-1α, a transcriptional factor, and its pro-survival functions are regulated by proline. The amino acid limits the HIF-1α degradation leading to upregulation of its transcriptional activity. Since PRODH/POX converts proline to glutamate and, then to α-ketoglutarate, it contributes to inhibition of HIF-1α transcriptional activity and limitation of cancer cell pro-survival properties [138].

It is a well-documented fact that the conversion of proline to P5C generates reactive oxygen species (ROS) or ATP [139]. The proline cycle is accompanied by the transfer of electrons into the mitochondria resulting in ROS generation [127]. Phang et al. showed that PRODH/POX contributes to the induction of apoptosis via mitochondrial and death receptor pathways leading to increased ROS generation [127]. The regulation of expression of PRODH/POX is driven by tumor suppressor protein p53, peroxisome proliferator-activated receptor γ (PPARγ), AMP-activated protein kinase (AMPK), phosphoinositide 3-kinase (PI3K), and an oncogene c-MYC through miR23b* [128]. In vivo study showed that overexpressed PRODH/POX favors inhibition of tumor growth [140] suggesting the strongly pronounced generation of P5C, as an intermediate for the TCA cycle metabolites, and ROS. It is known that overproduction of ROS causes intracellular damages, however, it has been found that ROS plays an important role as physiological signaling molecules under cellular stress. Thus, the role of the accelerated proline cycle may serve as a protective mechanism for maintaining redox balance including antioxidant enzymes (NADP+/NADPH and GSH/GSSG). Hoque et al. [141] reported that intracellular proline biosynthesis was stimulated upon supplementation with proline to the cell culture medium and, as a result, increased total GSH level. Proline has also been proposed as a scavenger of ROS and protein stabilizer [127]. Thus, an increased level of proline in several reports on reviewed gynecological and breast cancers could be associated with better prognosis and could be a potential biomarker. However, in these studies, there is no correlation between proline concentration and patient survival. Therefore, this gap needs to be filled to expand the knowledge about the relationship of proline level with clinico-pathological parameters.

**Figure 5:**
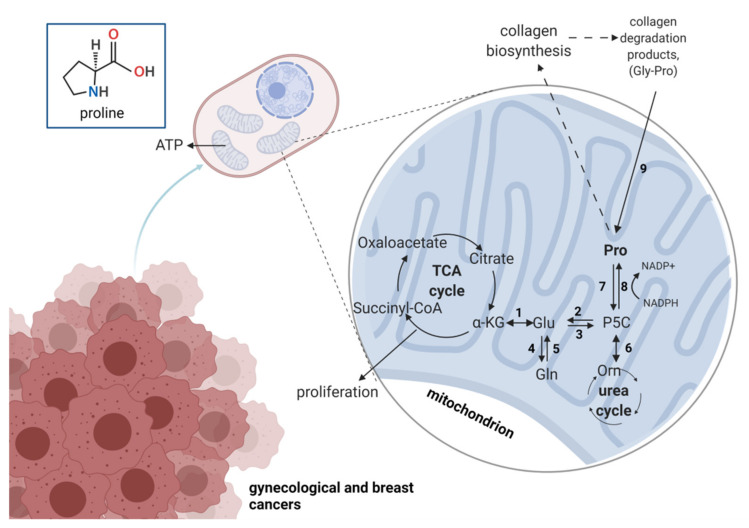
The overview on proline metabolism in gynecological cancers. Numbers represent enzymes involved in proline-relation reactions: 1—glutamate dehydrogenase; 2—P5C dehydrogenase; 3—P5C synthase; 4—glutamine synthase; 5—glutaminase; 6—ornithine aminotransferase; 7—proline dehydrogenase/oxidase; 8—P5C reductase; 9—prolidase; TCA, tricarboxylic acid. α-KG—alpha ketoglutarate; Glu—glutamic acid; Gln—glutamine; Gly—glycine; Orn—ornithine; P5C—pyrroline-5-carboxylate; Pro—proline. Created with Biorender.com accessed on 13 July 2021.

Since cancer cells exhibit an unlimited capacity for invading adjacent tissues, blood vessels, and distant organs, there is a need to inhibit metastases formation. Recently, it has been found that proline metabolism is involved in this process through the upregulation of PRODH/POX expression [142]. Another study showed upregulation of PYCR1 expression suggesting that cycling of P5C-proline is enhanced. Craze et al. [143] reported an increased PYCR1 copy number in BC. They also observed a higher level of proline-related protein deregulation such as ALDH18A1 and glutaminase in highly proliferative breast cancer cells. It indicates the glutamine-proline axis as a poor prognostic biomarker. Ding et al. [144] discovered that PYCR1 is overexpressed in BC and is positively correlated with tumor size, stage, and higher metastatic potential. In vitro study showed that knockdown of PYCR1 diminished the proliferative potential of breast cancer cells proliferation and sensitize the cells for chemotherapeutic drugs. The mechanism underlying reprogrammed metabolism in cancer cells remains largely unknown, however, upregulated proline-related enzymes allow cancer cells to produce ATP for survival. It seems interesting from a cancer therapy point of view to inhibit proline metabolism by targeting PRODH/POX and PYCR1 and interfering with metastasis formation. The overview on proline metabolism in gynecological cancers is presented in Figure 5.

## 8. Concluding Remarks

The presented review summarizes the available literature on changes in amino acid profiles in gynecological and breast cancers. It is evident that cancer growth and progression affect various metabolic pathways and thus alter the levels of certain amino acids in biological fluids and/or tissue. The studies of the metabolome of cancer patients explain the process of carcinogenesis and enable researchers to understand certain mechanisms of cancer progression. As amino acids are crucial for nucleotide synthesis, they could be promising cancer biomarkers. However, due to the complexity of metabolomics alterations it is most probable that a whole panel of substances, rather than a single compound, could serve as a discriminating tool. The potential applications of these markers or panels of markers are innumerable: from screening and early detection, through pre-treatment assessment of the disease type and risk-group stratification, predicting the response to chemotherapeutic agents, to monitoring the effects of treatment. Effective models based on the novel biomarkers could become powerful tools in the hand of clinicians and could contribute to early disease detection and individualization of treatment, hopefully positively affecting the prognosis of patients in the long term. Unfortunately, the results of various studies are sometimes inconclusive, and this is probably due to the dynamic and complex changes of amino acid levels in the body as well as many confounding factors. In should be noted that the studies discussed in the review employed different inclusion criteria for cancer patients and different control groups. Moreover, the research involved the analysis of various biological matrices that implemented various metabolomic methods and strategies offering different specificity and sensitivity. Further research in the field and multiple external validation studies are needed to establish the role of amino acid profiling in diagnosing gynecological and breast cancers. One of the crucial gaps that needs to be filled is elucidating proline anabolism and catabolism to better understand mechanism underlying reprogrammed metabolism in cancer cells and propose new cancer markers and targets for therapies.

## Figures and Tables

**Table 1:** Overview of metabolomic studies on endometrial, breast, ovarian, and cervical cancer and their results.

Reference	Disease	Design	Matrix	Method	Strategy	Differentiating Amino Acids	Validation ^1^
Ihata et al., 2014 [9]	Endometrial cancer	Endometrial cancer (*n* = 80); Gynecological benign diseases (*n* = 122); Healthy controls (*n* = 240)	Plasma	HPLC-MS/MS	Targeted	Asparagine (↑), glutamine (↑), histidine (↓), isoleucine (↑), leucine (↓), methionine (↓), ornithine (↑), phenylalanine (↓), proline (↑), serine (↓), tryptophan (↓), valine (↓)	Yes
Troisi et al., 2018 [10]	Endometrial cancer	Healthy subjects (*n* = 130); Endometrial Cancer (*n* = 118); Ovarian Cancer (*n* = 30); Benign endometrial disease (*n* = 10)	Serum	GC-MS	Untargeted	Homocysteine (↑), threonine (↓), valine (↓)	Yes
Gaudet et al., 2012 [11]	Endometrial cancer	Endometrial cancer(*n* = 250); Controls (*n* = 250)	Serum	FIA-MS/MS	Targeted	Valine (↑)	No
Shi et al., 2018 [12]	Endometrial cancer	Endometrial cancer (*n* = 46); Healthy controls (*n* = 46)	Serum	UHPLC-MS	Untargeted	Phenylalanine (↑)	No
Suzuki et al., 2018 [13]	Endometrial cancer	Endometrial cancer (*n* = 53); pre-surgery vs. post-surgery	Plasma	HPLC-MS/MS	Targeted	Citrulline (↓), histidine (↓), isoleucine (↑), tryptophan (↓), valine (↓)	No
Bahado-Singh et al., 2017 [14]	Endometrial cancer	Endometrial cancer (*n* = 56); Controls (*n* = 60)	Serum	^1^H-NMR; LC-MS/MS; FIA-MS/MS	Untargeted and targeted	Asparagine (↓), glutamate (↑), methionine (↓)	Yes
Audet-Delage et al., 2018 [15]	Endometrial cancer	Control women (*n* = 18); Type I endometrioid (*n* = 24); Type II serous carcinomas (*n* = 12)	Serum	UHPLC-MS/MS	Untargeted	Glycine (↓)	No
Strand et al., 2019 [16]	Endometrial cancer	Short survival (*n* = 20); Long survival (*n* = 20)	Plasma	LC-MS/MS; FIA-MS/MS	Targeted	Methionine sulfoxide (↓)	No
Altadill et al., 2017 [17]	Endometrial cancer	Endometrial cancer (*n* = 39); Healthy controls (*n* = 17)	Tissue	UHPLC-MS	Untargeted	Arginine (↓), glutamate (↓), phenylalanine (↓), tryptophan (↓)	Yes
Trousil et al., 2014 [18]	Endometrial cancer	Control group (*n* = 10); Endometrial cancer (*n* = 8)	Tissue	^1^H-NMR	Untargeted	Alanine (↑), leucine (↑), proline (↑), valine (↑), tyrosine (↑)	No
Cheng et al., 2019 [19]	Endometrial cancer	Endometrial cancer (*n* = 21); non-endometrial cancer (*n* = 33)	Cervicovaginal fluid	^1^H-NMR	Untargeted	Aspartate (↓), asparagine (↓), isoleucine (↓), phenylalanine (↓)	Yes
Zhou et al., 2010 [20]	Ovarian cancer	Ovarian cancer (*n* = 44); Healthy women or with benign condition (*n* = 50)	Serum	DART-MS	Untargeted	Alanine (↑), cystine (↑), glycine (↑), serine (↑), threonine (↑)	No
Hilvo et al., 2015 [21]	Ovarian cancer	Ovarian cancer (high grade) (*n* = 158); Benign ovarian tumors and healthy control (*n* = 100)	Serum, tissue	GC-MS	Untargeted	Alanine (↓), glutamate (↑), glutamine (↑), glycine (↑), methionine (↓), phenylalanine (↓), proline (↓), serine (↓), threonine (↓), tryptophan (↓), tyrosine (↓), valine (↓)	Yes
Garcia et al., 2011 [22]	Ovarian cancer	Ovarian cancer (early stage FIGO I/II) (*n* = 170); Healthy controls (*n* = 182)	Serum	^1^H-NMR	Untargeted	Alanine (↓), valine (↓)	Yes
Bachmayr-Heyda et al., 2017 [23]	Ovarian cancer	Ovarian cancer (high-grade serous) (*n* = 65); Healthy controls (*n* = 62)	Serum, ascites, tissue	LC-MS/MS; FIA-MS/MS	Targeted	Asparagine (↓), histidine (↓), lysine (↓), threonine (↓), tryptophan (↓)	Yes
Buas et al., 2016 [24]	Ovarian cancer	Ovarian cancer (serous) (*n* = 50); Benign ovarian tumors (serous) (*n* = 50)	Plasma	HPLC-MS	Targeted	Alanine (↓)	No
Ke et al., 2014 [25]	Ovarian cancer	Ovarian cancer (*n* = 140); Benign ovarian tumors/uterine fibromas (*n* = 308)	Plasma	UHPLC-MS	Untargeted	Histidine (↓), lysine (↓), phenylalanine (↓), tryptophan (↓)	Validation of previous research
Miyagi et al., 2017 [26]	Ovarian cancer	Ovarian cancer + borderline tumors (*n* = 80); Benign ovarian tumors (*n* = 97)	Plasma	HPLC-MS/MS	Targeted	Histidine (↓), isoleucine (↑), proline (↑), tryptophan (↓)	Validation of previous research
Zhang et al., 2012 [27]	Ovarian cancer	Ovarian cancer (*n* = 80); Benign ovarian tumors (*n* = 90)	Urine	UHPLC-MS	Untargeted	Tryptophan (↓)	Yes
Horala et al., 2021 [28]	Ovarian cancer	Ovarian cancer + borderline tumors (*n* = 44); Benign ovarian tumors (*n* = 62)	Serum	HPLC-MS/MS	Targeted	Aminoadipic acid (↓), asparagine (↓), citrulline (↓), cystine (↓), glutamine (↑), histidine (↓), isoleucine (↑), leucine (↑), phenylalanine (↑), threonine (↓), tryptophan (↓)	No
Plewa et al., 2017 [29]	Ovarian cancer	Ovarian cancer (*n* = 38); Benign ovarian tumors (*n* = 62); Healthy controls (*n* = 50)	Serum	HPLC-MS/MS	Targeted	Citrulline (↓), histidine (↓), lysine (↓), phenylalanine (↓), threonine (↓), tryptophan (↓)	No
Plewa et al., 2019 [30]	Ovarian cancer	Ovarian cancer (*n* = 26); Benign ovarian tumors (*n* = 25); Healthy controls (*n* = 25)	Serum	HPLC-MS/MS	Targeted	Citrulline (↓), histidine (↓)	Validation of previous research
Slupsky et al., 2010 [31]	Ovarian cancer	Ovarian cancer (*n* = 40); Healthy controls (*n* = 62)	Urine	^1^H-NMR	Untargeted	Alanine (↓), asparagine (↓), isoleucine (↓), leucine (↓), valine (↓)	No
Ahn et al., 2020 [32]	Ovarian cancer	Ovarian cancer (*n* = 10); Healthy controls (*n* = 10)	Plasma	UHPLC-MS/MS; FIA-MS/MS	Targeted	Ornithine (↓), tryptophan (↓)	No
Wang et al., 2021 [33]	Ovarian cancer	Ovarian cancer (*n* = 39); Healthy controls (*n* = 31)	Serum	UHPLC-MS/MS	Targeted	Asparagine (↑), glutamine (↑), methionine (↑)	Yes
His et al., 2019 [34]	Breast cancer	Invasive breast cancer (*n* = 1624); Control group (*n* = 1624)	Plasma	LC-MS/MS; FIA-MS/MS	Targeted	Arginine (↓), asparagine (↓)	No
Eniu et al., 2018 [35]	Breast cancer	Breast cancer (*n* = 30); Healthy controls (*n* = 26)	Serum	UHPLC-MS	Targeted	Alanine (↓), arginine (↓), glutamine (↓), isoleucine (↓), leucine (↓), tyrosine (↓)	No
Mitruka et al., 2020 [36]	Breast cancer	Breast cancer (*n* = 10); Healthy controls (*n* = 12)	Nails	HPLC-MS	Untargeted	Histidine (↓), phenylalanine (↓), tryptophan (↓), tyrosine (↓)	No
Shen et al., 2013 [37]	Breast cancer	Breast cancer (*n* = 60); Healthy controls (*n* = 60)	Plasma	UHPLC-MS/MS; GC-MS	Untargeted	Alanine (↓), glutamine (↓), histidine (↓), methionine (↓), proline (↓)	No
Budczies et al., 2013 [38]	Breast cancer	Estrogen receptor positive (ER+) (*n* = 204); Estrogen receptor negative (ER−) (*n* = 67)	Tissue	GC-MS	Untargeted	Alanine (↑), glutamate (↑), glutamine (↓)	Yes
Cala et al., 2018 [39]	Breast cancer	Breast cancer (*n* = 29); Healthy controls (*n* = 29)	Plasma	GC–MS; LC-MS; ^1^H-NMR	Untargeted	Alanine (↑), cystine (↓), isoleucine (↓), threonine (↓), tryptophan (↓)	No
Miyagi et al., 2011 [40]	Breast cancer	Breast cancer (*n* = 196); Healthy controls (*n* = 976)	Plasma	HPLC-MS/MS	Targeted	Alanine (↑), glutamine (↓), glycine (↑), histidine (↓), ornithine (↑), phenylalanine (↓), proline (↑), serine (↑), tryptophan (↓), tyrosine (↓)	No
Moore et al., 2021 [41]	Breast cancer	Breast cancer (*n* = 782); Control group (*n* = 782)	Serum	UHPLC-MS	Untargeted	Cystine (↑)	Validation of previous research
Cao et al., 2015 [42]	Breast cancer	Breast cancer (*n* = 20); Healthy controls (*n* = 50)	Serum	FIA-MS/MS	Targeted	Tryptophan (↑)	No
Xie et al., 2015 [43]	Breast cancer	Breast cancer (*n* = 35); Control group (*n* = 35)	Plasma, serum, tissue	HPLC-MS; GC-MS	Untargeted	Aspartate (↓)	Yes
Wang et al., 2016 [44]	Breast cancer	Breast cancer (*n* = 258); Control group (*n* = 159)	Dried blood spot	DIMS	Targeted	Asparagine (↓), cystine (↓), histidine (↓), homocysteine (↓), lysine (↓), proline (↓), tyrosine (↑), tryptophan (↓)	Yes
Jasbi et al., 2018 [45]	Breast cancer	Breast cancer (*n* = 102); Healthy controls (*n* = 99)	Plasma	UHPLC-MS/MS	Targeted	Proline (↓)	No
Yuan et al., 2018 [46]	Breast cancer	Breast cancer (*n* = 80); Healthy controls (*n* = 100)	Plasma	LC-MS/MS; FIA-MS/MS	Targeted	Alanine (↓), asparagine (↓), glutamine (↓), histidine (↓), leucine (↓), lysine (↓), methionine (↓), ornithine (↓), phenylalanine (↓), threonine (↓), tryptophan (↓), tyrosine (↓), valine (↓)	Yes
Li et al., 2020 [47]	Breast cancer	Breast cancer (*n* = 31); Healthy controls (*n* = 31)	Serum	HPLC-MS	Untargeted	Leucine (↑), proline (↑), threonine (↑), tyrosine (↑), valine (↑)	Yes
Khan et al., 2019 [48]	Cervical cancer	Cervical cancer (*n* = 60); Healthy controls (*n* = 69); CIN1 (*n* = 55); CIN2/3 (*n* = 42)	Plasma	UHPLC-MS	Untargeted	Aspartate (↑), glutamate (↑), proline (↑)	No
Abudula et al., 2020 [49]	Cervical cancer	Negative controls (*n* = 11); Cervical cancer (*n* = 21)	Tissue	^1^H-NMR	Untargeted	Alanine (↓), isoleucine (↓), methylproline (↓), phenylalanine (↓), tyrosine (↓)	Yes
Yang et al., 2017 [50]	Cervical cancer	Negative controls (*n* = 149); Cervical cancer (*n* = 136)	Plasma	UHPLC-MS	Untargeted	Lysine (↓)	No
Chen et al., 2013 [51]	Cervical cancer	Negative control (*n* = 23); Cervical cancer (*n* = 22)	Urine	LC-MS/MS	Untargeted	Tryptophan (↓), tyrosine (↓)	No
Hasim et al., 2012 [52]	Cervical cancer	Negative control (*n* = 38); Cervical cancer (*n* = 38)	Plasma	^1^H-NMR	Untargeted	Alanine (↓), isoleucine (↓), leucine (↓), valine (↓)	No

^1^ Internal validation of the constructed discriminating models was not considered. Abbreviations: DART-MS, direct analysis in real-time mass spectrometry; DIMS, direct infusion mass spectrometry; FIA-MS/MS, flow-injection tandem mass spectrometry; GC-MS, gas chromatography-mass spectrometry; ^1^H-NMR, proton nuclear magnetic resonance; HPLC-ESI-MS, high-performance liquid chromatography-electrospray ionization-mass spectrometry; HPLC-ESI-MS/MS, high-performance liquid chromatography-electrospray ionization-tandem mass spectrometry; LC-ESI-MS/MS, liquid chromatography-electrospray ionization-tandem mass spectrometry; UHPLC-ESI-MS, ultra-high-performance liquid chromatography-electrospray ionization-mass spectrometry.

## Data Availability

Data sharing not applicable.
